# Grouping and Sponsoring Centric Green Coverage Model for Internet of Things

**DOI:** 10.3390/s21123948

**Published:** 2021-06-08

**Authors:** Vinod Kumar, Sushil Kumar, Rabah AlShboul, Geetika Aggarwal, Omprakash Kaiwartya, Ahmad M. Khasawneh, Jaime Lloret, Mahmoud Ahmad Al-Khasawneh

**Affiliations:** 1 School of Computer and Systems Sciences, Jawaharlal Nehru University (JNU), New Delhi 110067, India; vinod_k@dtu.ac.in (V.K.); skdohare@mail.jnu.ac.in (S.K.); 2 Computer Science Department, Faculty of Information Technology, Al al-Bayt University, Mafraq 25113, Jordan; rabahshboul@aabu.edu.jo; 3 School of Science & Technology, Clifton Campus, Nottingham Trent University, Nottingham NG11 8NS, UK; omprakash.kaiwartya@ntu.ac.uk; 4 Department of Mobile Computing, Amman Arab University, Amman 11953, Jordan; a.khasawneh@aau.edu.jo; 5 Integrated Management Coastal Research Institue, Universitat Politecnica de Valencia, 46022 Valencia, Spain; jlloret@dcom.upv.es; 6 School of Computing and Digital Technologies, Staffordshire University, Stoke ST4 2DE, UK; 7 Faculty of Computer & Information Technology, Al-Madinah International University, Kuala Lumpur 57100, Malaysia; mahmoud@outlook.my

**Keywords:** energy efficiency, green computing, lifetime maximization, internet of things

## Abstract

Recently, green computing has received significant attention for Internet of Things (IoT) environments due to the growing computing demands under tiny sensor enabled smart services. The related literature on green computing majorly focuses on a cover set approach that works efficiently for target coverage, but it is not applicable in case of area coverage. In this paper, we present a new variant of a cover set approach called a grouping and sponsoring aware IoT framework (GS-IoT) that is suitable for area coverage. We achieve non-overlapping coverage for an entire sensing region employing sectorial sensing. Non-overlapping coverage not only guarantees a sufficiently good coverage in case of large number of sensors deployed randomly, but also maximizes the life span of the whole network with appropriate scheduling of sensors. A deployment model for distribution of sensors is developed to ensure a minimum threshold density of sensors in the sensing region. In particular, a fast converging grouping (FCG) algorithm is developed to group sensors in order to ensure minimal overlapping. A sponsoring aware sectorial coverage (SSC) algorithm is developed to set off redundant sensors and to balance the overall network energy consumption. GS-IoT framework effectively combines both the algorithms for smart services. The simulation experimental results attest to the benefit of the proposed framework as compared to the state-of-the-art techniques in terms of various metrics for smart IoT environments including rate of overlapping, response time, coverage, active sensors, and life span of the overall network.

## 1. Introduction

An Internet of Things (IoT) centric wireless sensor network has many interpretations in different contexts [1]. For a naïve user, it is like a network of tiny sensor nodes, which perceives the environment and provides data for enabling smart services with data analysis. For a researcher, it is a network of tiny sensors, but they are to be made intelligent enough for smart service oriented prolonged sensing [2]. In IoT centric networking, there is a tradeoff between energy saving and coverage centric connectivity, which needs to be optimized for better network performance and longer network lifetime [3]. In contemporary time, wireless sensor networks were working under the push factor from advancing microelectromechanical technology where information flows towards sensor to service only. However, in IoT centric smart services, they work on pull factors where information flows in both directions from sensor to service as well as service to sensor for increasing real-time smart service usability [4]. The basic technology for sensing includes radio wave sensing, infrared sensing, radar sensing, seismic sensing, etc. However, rapidly changing technology is making sensors smarter and more accurate in resolution and sensing, which makes green computing technologies more significant in current scenarios [5,6].

For enabling IoT centric smart services, computing and communication capability enabled tiny sensors are deployed in the sensing environments [7]. The service centric deployment is based on two major strategies including preplanned for non-hostile, and random for hostile environments. In the first, the tiny sensor nodes are deployed in well-calculated and preplanned locations and hence this deployment could be optimized [8]. For example, video sensors in buildings for security surveillance [9], roadside sensing units for traffic monitoring [10,11,12], and sensors on patient body and hospital infrastructure for diagnostics are some examples of measured IoT deployment scenarios [13,14]. However, in the age of smart service, different service providers deploy their networks resulting in coverage redundancy and interference. In the second, the tiny sensors are deployed in random locations without well-established accessibility in the hostile region [15]. Therefore, in IoT centric networking for smart services, both types of deployment scenarios have sensor redundancy due to the service centric performance guarantee with a larger number of sensors in the region of interest [16]. However, the redundancy of sensors could be utilized positivity via various potential approaches including virtualization for reliability [17], sponsoring aware grouping, and scheduling with job allocation [18]. These approaches are well established for enabling application domain centric environments. However, these approaches could be improved for smart service centric green computing environments focusing on grouping optimization and coverage sponsorship.

Towards this end, this paper proposes a grouping and sponsoring centric green computing framework for enabling Internet of Things (GS-IoT). The framework focuses on optimal grouping of sensors and sponsoring coverage among neighbours in the network for prolonging the lifetime of smart services. In particular, the potential technical contributions of the paper can be summarized in following major folds:

- Firstly, a network model for coverage redundancy management of sensors is presented considering the smart campus centric IoT environment.
- Secondly, a distributed fast converging grouping method is developed for optimal overlapping of active sensor management in coordinated network scenarios.
- Thirdly, a sponsoring aware sectorial coverage model is derived focusing on local group knowledge about redundant sensors and their coverage ranges.
- Finally, comparative performance evaluation of the proposed framework is carried out focusing on analytical, simulation, and hardware-based implementations and critical result discussions considering some recent literature in IoT.

The rest of the paper is organized as follows: Section 2 critically reviews related literature on green computing for IoT. Section 3 presents the details of the proposed GS-IoT framework. Experiments and critical result analysis are discussed in Section 4, followed by conclusions presented in Section 5.

## 2. Related Work

The problem of providing effective coverage has been solved in different ways in the literature—for example, cover set approach, redundancy approach, sectorial sensing, and many more. To ensure coverage in a randomly distributed IoT enabled sensor network is rather a tough task as compared to a pre-planned network environment. In [19], authors have suggested a cover set based solution for a coverage problem. It is intended to cover all the targets by a maximum number of possible cover sets. The cover set problem is NP-complete. A heuristic is applied to find a working solution. A similar target coverage problem is solved in [20]. This solution is also based on the heuristic approach for covering discrete set of points. The author has proposed a power aware coverage maximizing solution in [21]. This solution is also based on the discrete cover sets which cover a countable localized set of targets. Since finding a maximum number of cover set is an NP-complete problem, these solutions are an approximate solution to the problem. In the process, they are a bit cumbersome and energy-consuming.

In [22], authors have proposed a solution for ensuring k-coverage in the area. This type of coverage ensures reliability and robustness of networks. This is also an algorithmic approach that runs in polynomial time. Approach in this work is to divide the whole region in sub regions and then ensure k-coverage of these sub regions. They claim that the algorithm works efficiently. A multi-objective free search algorithm is presented in [23]. This algorithm ensures and establishes a balance between contradictory objectives in a wireless sensor network. These contradictory objectives are maximizing the coverage, optimizing the energy consumption, and many more. In [24], authors have proposed a scheduling algorithm for increasing the life expectancy of wireless sensor networks. At the same time, this algorithm ensures the coverage of discrete set of points lying over field. The algorithm is based on connected coverage methodology. In [25], authors have suggested a method for coverage of a number of discrete points through sectorial sensing. They have formulated maximum coverage with a minimum sensors problem (MCMS), and they solved MCMS applying exact integer linear programming. However, it is a centralized algorithm and, therefore, is not suitable for large sensor networks.

A detailed survey about the coverage and associated sensing model is presented in [26]. In this review article, the author has presented an elaborate account of the bits of the coverage problem. In [27], a cover set methodology is adopted for covering a discrete set of points in order to maximize the lifetime of the network. This work also turned out to be a good heuristic-based approach for solving the problem of both coverage and life expectancy. Almost the same type of work has been done in [28]; however, it is based on different heuristics. These heuristics-based solutions are efficient enough in their respective domain. However, they suffer from the problem of suitability of underlying assumptions for the heuristic itself. In Ref. [29], authors have proposed a very novel model for coverage rate calculation. This objective is achieved through a distributed exact coverage rate calculation and distributed probabilistic coverage rate calculation. This approach increases reliability in data delivery by a sensor network. In [30], authors have suggested a scheme to set off redundant sensors. This model is implemented over traditional energy-based protocol for wireless sensor networks. It simply shows that keeping redundant nodes off, one can increase the life span of the network. In [31], a scheme has been proposed of how controlled overlapping can be introduced to save energy in greater interest of the network.

A green computing framework for IoT considering interference (GC-IoT) as a major modeling parameter has been suggested [32]. A shortest path strategy with a lesser number of forwarding transmitter nodes has been applied for avoiding interference among neighbouring nodes during multi-hop transmission. Mathematical modeling of energy consumption and queueing time have been derived for better understanding the green communication in the approach. Although the interference aware energy modeling reduced energy consumption up to some level for longer network lifetime, overlapping of coverage areas among sensor nodes results in the wastage of energy as sensor nodes might not interfere each other’s communication while still overlapping coverage with each other. A similar green computing framework for wireless sensor networks (GC-WSN) has been suggested considering mobility prediction and relocation of nodes in a tree-oriented architecture for better energy utilization during communications [33]. A tree-based network architecture has been developed considering energy consumption level in different communication paths. The sensor nodes have been virtually shifted to different levels of the tree-based network for better energy utilization. The network lifetime optimization problem has been mathematically modelled along with algorithms for balancing the nodes on the three network-based architectures considering node switching. Although the balanced tree-based architecture has been claimed to be increasing the overall lifetime of the network by appropriate path selection on the tree network. However, in case of IoT centric network architecture with dense environments, its applicability is limited considering the size of the tree and managing node switching on that large tree network [34]. An IoT technology centric critical review [35] and green computing in IoT [36,37] have been quite significant developments such as enabling intelligent architectures for smart use cases of IoT. Towards this end, we propose a network architecture to ensure minimal overlap of coverage. For this purpose, we have employed sectorial sensing model for sensing in selected sectors and, if necessary, keep the sensors off. This approach for solving coverage problem is fault tolerant. In the next section, we attempt to overcome the problem of coverage in an adaptive manner, which is potential for IoT centric smart services. We have modeled the distribution of sensors in the field of interest. This is necessary, as we have to ensure a definite sensor density in every part of sensing field. We propose an adaptive and dynamic algorithm for self-organization and scheduling of these sensors for IoT centric smart services.

## 3. Grouping and Sponsoring Centric Green Computing for IoT

### 3.1. Network Model

**Figure 1:**
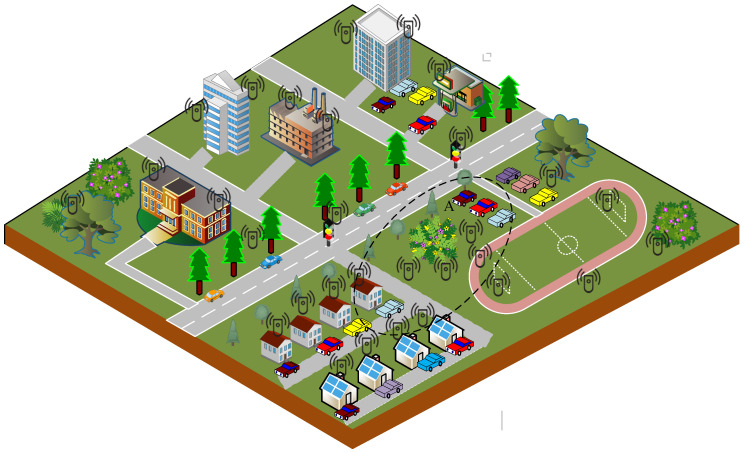
Smart campus environment with densely deployed sensors for round the clock information gathering and surveillance.

The problem of finding a maximum number of a cover set for a universal set of sensors in IoT centric networking is NP-Complete. Many solutions in the literature regarding this problem have been worked out for target coverage. All of these solutions without any exceptions are based on heuristics. These are approximate solutions that have their own discrepancies. These solutions are centralized in nature which is less scalable. However, to maximize the life span of IoT centric networking for smart services, we need to effectively schedule the sensors. For this purpose, we model our problem in a totally different way focusing on densely deployed smart service in IoT environments such as in the smart campus in Figure 1. The motivation for present work lies in the optimal usage of sensor energy considering network knowledge aspects of the IoT centric sensor network. The IoT enabled network for smart services works in self-organizing fashion and hence an adaptive, dynamic, and fault tolerant algorithm is needed for its proper functioning. Although researchers have worked in varying dimensions of these problems, work can be done to achieve greater efficiency in green computing dimensions. The smart service centric IoT deployment with a sensing range of sensors is invariably circular in shape, hence coverage overlapping is inevitable in the smart environments. This overlapping results in the wastage of the energy of tiny sensors. Through the sectorial sensing model of sensors, we can achieve less overlapped coverage. If we could develop an efficient algorithm to ensure less overlapping, non-overlapping coverage not only guarantees a sufficiently better coverage in case of a large number of sensors deployed randomly, but also maximizes the life span of smart service centric IoT networks if sensors are scheduled properly with coverage sponsorship management. We want to clarify that we do understand the resource constrained centric sensors and sensor attached devices. However, here in our proposal, our assumption of a coordinated operating framework among a group of sensors is achievable and a realistic assumption, which we have proved with simulation and hardware experiments. Furthermore, there are growing advancements in sensing technology day by day, and the resource constrained sensors are becoming intelligent and are able to make smart decisions for different IoT use cases.

Being motivated from all of the above smart environment aspects, we intend to find a new cover set scheme for area coverage in smart service centric IoT environments. We divide the entire smart sensing region into small regions of sensing and cover it through a group of sensors. These formed groups are changed after a fixed time interval so that we can bring randomization in energy consumption. A mathematical formulation for this problem is derived. Let S denote a subset in $R^{2},$ and C is a set of subsets in S. For a∈C, let | a| denote the size of a. S is the area of sensing region. The sensing region is partitioned in small regions which are mutually disjoint. It is assumed that they contain at least three sensors. These three sensors must be connected to each other, and they should provide full coverage to the area in which they fall. $C=\left\{\;a_{i}\right\}\;for\;i=1,2\ldots n$, where C denotes the set of those regions and $a_{1}\cup^{\;}a_{2}\ldots \cup^{\;}a_{n}=S$ and$\;a_{1}\cap^{\;}a_{2}\ldots \cap^{\;}a_{n}=\emptyset$. A type of such region may be coverage provided by an individual sensor. To formulate this problem, it is assumed that sensors are following Poisson distribution statistically. There are N sensors deployed in a square sensing region with side length R. $X\left(a_{i}\right)$ is the number of sensors in the region$\;a_{i}\;for\;i=1,\;2,\ldots ,n$. $X_{i}$ is a Poisson random variable with intensity λ if the following assumptions are made. For all $a_{i},$ $X\left(a_{i}\right)$ follows a Poisson process with intensity λ which depends only on $\left|a_{i}\right|$. All $X\left(a_{i}\right),\;i=1,\;2,\;\ldots ,\;n$ are independent. *P* ($X\left(a_{i}\right)\geq 1)=\lambda \left|a_{i}\right|+o\left(\left|a_{i}\right|\right)$). The probability of overlap is zero in the set. i.e., $\underset{\left|a_{i}\right|\to 0}{\lim}\frac{P\left(X\left(a_{i}\right)\geq 1\right)}{P\left(X\left(a_{i}\right)=1\right)}=1$. If all the above axioms are followed, we have:

(1) $$P\left(X\left(a_{i}\right)=k\right)=\frac{e^{-\lambda \left|a_{i}\right|}{\left(\;\lambda \left|a_{i}\right|\right)}^{k}}{k!}$$

Now, to specify the region, we need to know what kind of distribution is followed by the sensors inside each region$\;a_{i}$. For this, we consider that this is following Binomial Distribution. It can be mathematically derived as follows.

Considering smart campus sensor deployment in Figure 1, it shows sensor points following the Poisson process. Let us calculate the distribution pattern of sub region A:

$$P\left(X\left(B\right)=1|X\left(A\right)=1\right)=\frac{\left|B\right|}{\left|A\right|},\;\mathrm{where}\;B\subset A$$

This consideration can be validated via mathematical derivation given in the following probabilistic modeling steps:

$$P\left(X\left(B\right)=1|X\left(A\right)=1\right)=\frac{P\left(X\left(B\right)=1,X\left(A\right)=1\right)}{P\left(X\left(A\right)=1\right)}\;P\left(X\left(B\right)=1|X\left(A\right)=1\right)=\frac{P\left(X\left(B\right)=1,X\left(A\cap^{\;}B^{c}\right)=1\right)}{P\left(X\left(A\right)=1\right)}$$

From (1), we have

$$P\left(X\left(B\right)=1|X\left(A\right)=1\right)=\frac{\lambda \left|B\right|e^{-\lambda \left|B\right|}e^{-\lambda \left|A\cap^{\;}B^{c}\right|}}{\lambda \left|A\right|e^{-\lambda \left|A\right|}}\;P\left(X\left(B\right)=1|X\left(A\right)=1\right)=\frac{\lambda \left|B\right|e^{-\lambda \left|A\right|}}{\lambda \left|A\right|e^{-\lambda \left|A\right|}}$$

(2) $$P\left(X\left(B\right)=1|X\left(A\right)=1\right)=\frac{\left|B\right|}{\left|A\right|}$$

Now, if we generalize this result for X(A)=m and X(B)=l, where *l* = 0, 1, … *n*, we can write this as follows:

(3) $$P\left(X\left(B\right)=l|X\left(A\right)=m\right)=m_{∁}{}_{l}{\left(\frac{\left|B\right|}{\left|A\right|}\;\right)}^{l}\;{\left(\frac{\left|B\right|}{\left|A\right|}-1\;\right)}^{m-l}\;$$

Let r be the sensing range of a sensor. We must ensure at least two sensors in the sensing range of each sensor. For this, sub region$\;a_{i}$‘s are chosen in such a way that they contain at least three sensors connected to each other. From Equation (3), we have

(4) $$P\left(X\left(B\right)=3|X\left(A\right)=3\right)=m_{∁}{}_{l}{\left(\frac{\left|B\right|}{\left|A\right|}\;\right)}^{l}\;{\left(\frac{\left|B\right|}{\left|A\right|}-1\;\right)}^{m-l}=1\;\Rightarrow \;\frac{\left|B\right|}{\left|A\right|}=1\;\;\Rightarrow \;\left|B\right|=\left|A\right|$$

Equation (4) is true if B∈A, but if the sensor is placed on the opposite side of the boundary in region A, this can not guarantee the connectedness. Hence, to guarantee this, the radius of a sub region must be half the sensing range. Now, we assume that$\;a_{i}=\pi {\left(\frac{r}{2}\right)}^{2},\;i=1,\;2,\;\ldots ,\;n$. Thus, Equation (1) can be expressed as

$$P(X\left(\pi {\left(r/2\right)}^{2}\right)=k)=\frac{e^{-\lambda \pi {\left(\frac{r}{2}\right)}^{2}}{\left(\lambda \pi {\left(\frac{r}{2}\right)}^{2}\right)}^{k}}{k!}$$

We denote this probability by P(k) for l=0,1,…,N as

(5) $$P\left(X\left(\pi {\left(r/2\right)}^{2}\right)\geq 3\right)=1-P\left(0\right)-P\left(1\right)-P\left(2\right)\;$$

and the total number of sensors can be expressed as:

(6) $$N=\lambda \cdot R^{2}$$

### 3.2. Fast Converging Grouping

**Figure 2:**
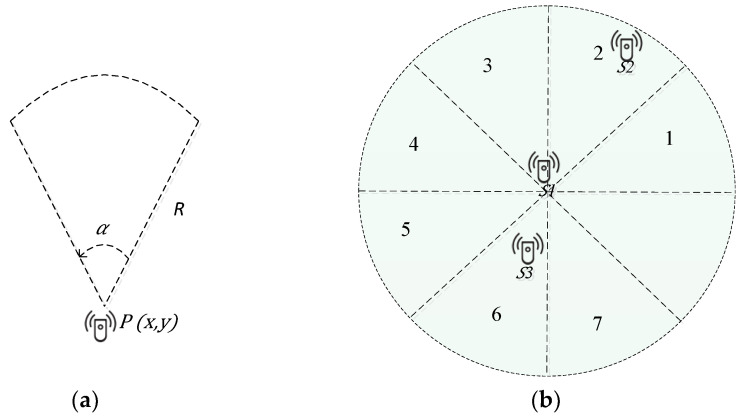
Smart environments, (**a**) angular sensing; (**b**) angular transmitting in in the region.

Towards enabling GS-IoT framework, the Fast Converging Grouping (FCG) algorithm is developed focusing on angular sensing and transmission in tiny sensors’ centric IoT environments. Considering Figure 2a, P(x, y) is the position of a sensor node S1 in smart service centric environments. It has the sensing range r. Offset angle α is an angle in which the sensor senses its environment at a particular time where values of α belong to {45, 90,..., 360} in degrees. The model of sectorial sensing is characterized by (*P*, α, r). Each sector in this sensing model is numbered from 1 to 8, and the sensor can start sensing through any sector or number of sectors, but they should be contiguous. We are presenting a model of transmitter which can take eight possible orientations. It transmits in sectors; otherwise, it will work like an omnidirectional transmitter. For example, in Figure 2b, node S1 can transmit packets in any of its different sectors one at a time. This model is used to detect any other node which falls under the transmission range of a particular node in a given sector. It is assumed that a node always starts from sector 1 to all possible orientations. For example, in Figure 2b, S2 is the node to be detected in the sector 2, and node S3 is to be detected in sector 6 of node S1. The Fast Converging Grouping (FCG) is a self-organizing algorithm for sensor nodes. Each sensor node looks to form its own group of three sensors with disjoint formed groups.

We completely understand that serverless and Function as a Service (FaaS) computing architecture are significant for enabling scalable IoT services [38]. We want to highlight that the proposed grouping functionality in the GS-IoT framework is technically based on quite similar assumptions. In the fast coverage grouping function presented in Algorithm 1, a group of deployed sensors coordinate with each other for providing efficient coverage without the need for any server like guiding framework.

**Definition** **1.** *Group* $G_{i}\;i=1,2,\ldots t$ *in the sensor network is a list of three sensor nodes that have following criteria:* $d\left(S_{1},\;S_{2}\right)$ *is the distance metric on* $R^{2}$ *. Any two sensors* $S_{1},\;S_{2}\;\in G_{i}$ *are connected if* $\;d\left(S_{1},\;S_{2}\right)\;\leq r$ *. At least two pairs of sensors in* $G_{i}$ *must be connected. To form a group, each sensor starts communicating to its neighbours to join the group in the smart environments.*

**Definition** **2.** *Two sensors *$S_{1}$*(x, y), and *$S_{2}$*(u, v) are in the neighbourhood of each other, if *${\left(x-u\right)}^{2}+{\left(y-v\right)}^{2}\leq r^{2}$.

**Figure 3:**
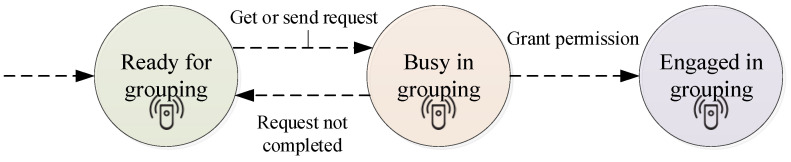
The three states of a sensor in smart environments.

We have a specific way to model this task. Each sensor in its slot sends a *hello-req* packet sector wise according to the proposed transmitting model. On the receipt of the *hello-req* packet, a sensor can respond by the *hello-rep* packet. Minimal transmission power is to be used to transmit packets up to sensing r only. Sensors send *hello-req* packets in the sectors to check the presence of other sensor nodes in the range. These packets carry information containing sector number and identity of senders. Receiving sensors reply with sending their identity, received power of transmission (P), status, sector, and identity of the sender node in a *hello-rep* packet. The status field of a sensor signifies whether this sensor is ready to be a part of this group. Sensors can be in three states (Figure 3).

To compute the distance between sender and receiving sensors, a node sends received power in a *hello-rep* packet. The computed distance between a pair of sensor nodes is used to minimize overlapping in FCG. This is how nodes collect information about their neighbours, e.g., Figure 2b, sensor S_1_ knows about the presence of node S_2_ in sector 2 and node S_3_ in sector 5. In the aforementioned paragraph, we have noted that a node sends a *hello-req* packet as soon as it gets a chance to do so. We derive a mathematical expression to compute the chance assuming that the waiting time follows an exponential distribution. The random variable Y represents the waiting time to start sending a *hello-req* packet. Each sensor will derive the value of Y independently and wait for a specified time before sending a *hello-req* packet. A sensor could be in three distinct states: *ready, busy,* and *engaged*. A sensor in *ready* state is free to join any group. If a sensor is a part of a group, it is designated as *engaged*. A sensor in a *busy* state has either received a *hello-req* from any one of its neighbours or is waiting for a *hello-rep* packet. Being in a ready state if the sensor gets a request from neighbour, it will respond to this request after the lapse of waiting time Z. The random variable Z represents waiting time before sending a *hello-rep* packet.

The following function $F_{Y}\left(y\right)$ represents a distribution of waiting time:

(7) $$F_{Y}\left(y\right)=1-\exp\left(-\gamma y\right)\;\forall \;y\geq 0$$

**Figure 4:**
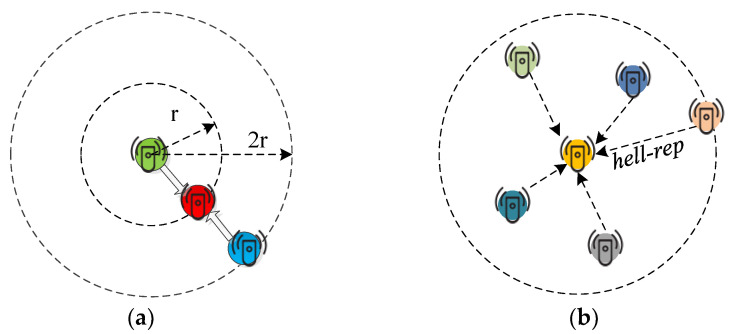
The interference scenario, (**a**) collision of grouping requests; (**b**) collision of grouping reply.

It depends on rate parameter γ which in turn depends on the expected number of nodes in an area of$\;\pi {\left(2r\right)}^{2}$. It is possible that a sensor receives two or more requests at the same time as depicted in Figure 4a,b. Since sensors follow the Poisson distribution in their appearance in the region, the expected value for this distribution is the expected number of sensors in area $\pi {\left(2r\right)}^{2}$ is given by $4\lambda \pi r^{2}.$ The total number of sensors are *N* and, at any time, *N*/$\;4\lambda \pi r^{2}$ collision free requests can be generated. Thus, the rate parameter γ can be expressed as given by Equation (8):

(8) $$\gamma =\frac{4\lambda \pi r^{2}}{N}$$

The *cdf* for waiting time for *hello-req* is given by:

(9) $$W_{Y}\left(t\right)=1-e^{-\frac{4\lambda \pi r^{2}t}{N}}\;\forall \;t\geq 0$$

where t ϵ [0, T], and this interval depends on the total number of nodes and communication time. Similarly, we can find waiting time for *hello-rep* (in area$\;\pi r^{2}$ shown in Figure 4b):

(10) $$W_{Z}\left(t\right)=1-e^{-\frac{\lambda \pi r^{2}t}{N}}\;\forall \;t\geq 0$$

The algorithm FCG for the forming group by the sensors is presented below. FCG runs for a certain time so that a sufficient number of groups are formed to cover the sensing area.
**Algorithm 1: Fast Converging Grouping (FCG)**j∈*N: a sensor in sensing region.* $K\in M_{j}$: *set of neighbouring sensors of node j.*b←number of Hellorep packets with status busy y←number of Hellorep packets with status ready e←number of Hellorep packets with status engaged ***begin:*** ***wait****(node j waits for time which is given by*$\;W\;\leftarrow 1-e^{-\frac{4\lambda \pi r^{2}t}{n}}$)***if** (hello-req from other sensor)* *send hello-rep packet after waiting for time*$\;W\;\leftarrow 1-e^{-\frac{\lambda \pi r^{2}t}{n}}$*set status busy* ***wait** (for confirmation for a certain time)* ***if**(no confirmation)* *set status ready* ***else*** *set status engaged* ***end if*** ***end wait*** ***end if*** ***end wait*** ***if** (list of members have less than three members) **set status busy* ***for** (sectors 1 to 8)* *sends hello-req packet* ***end for*** ***wait** (for a definite time for response)* *store all hello-rep packets* ***end wait*** ***if** (y >0)* Select sensors randomly to complete the group /*binomial distribution*/ *Send confirmation for group membership to select sensors* *Nodes which are not included go in ready state after waiting for a certain time. ****end if*** ***if** (group is not complete)* *go to 7****end if*** ***end begin***

### 3.3. Sponsoring Aware Sectorial Coverage

**Figure 5:**
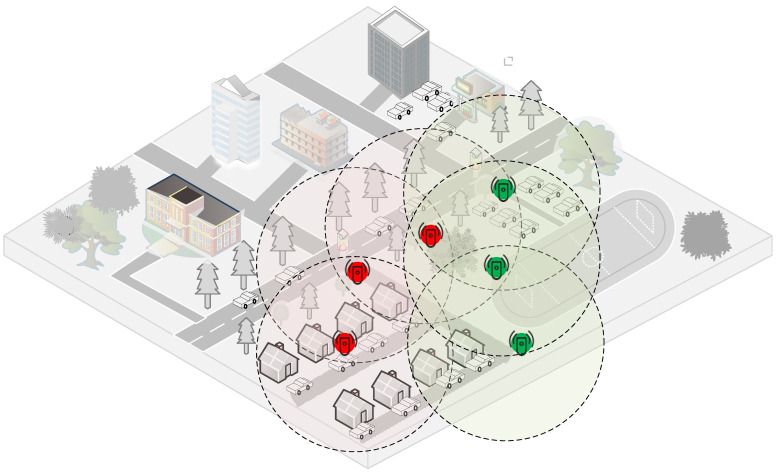
Smart IoT environment depiction of two groups of sensors which sponsors coverage (red) for each other (green).

The Sponsoring aware Sectorial Coverage (SSC) is a cooperation centric energy optimization among neighbouring sensors in the smart service centric IoT environments. It operates in the second stage of GS-IoT framework after the creation of groups of sensors at the first stage in the FCG technique. In each group, sensors have the information about other members including which sector they lie in and how far they are situated from each other. Figure 5 depicts two such groups. In this figure, red color sensors form one group and green color sensors form the other group. Now, sensors have to set their direction according to other sensors that are part of the group and also according to neighbouring sensors. Here, we want to highlight that, in the proposed GS-IoT framework, sensor nodes are not always in listening mode for enabling coordinated working framework. Once the fast coverage grouping is carried out, an acceptable and coordinated workflow is communicated among sensors, and component sensors follow the workflow subsequently resulting in switching off by group members in the coordinated manner. This workflow-oriented duty cycle management leads to the considerable amount of energy saving by providing coordinated coverage in the IoT environment.

**Figure 6:**
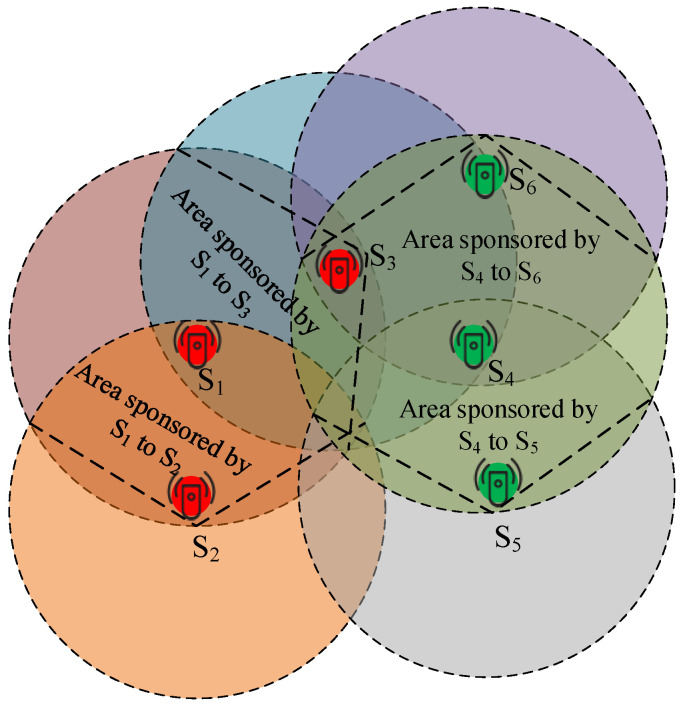
Group of sensors using coverage sponsor centric scheduling for non-overlapping coverage.

In each group, sensors set their priority for sensing in a particular area. Let us take the case of three red sensors in Figure 6 which are depicted by sensors - green, and red in Figure 6. Sensors in a group sense in rounds. In the first round, a sensor sponsors sensing coverage to other sensors, and the recipient becomes a sponsor in the next round. In this way, overlapping of the sensing region is avoided (cf. Figure 6). For example, the blue and red sensors sponsor coverage to each other’s.

**Definition** **3.** *A sensor,*I*, is said to be a coverage sponsoring sensor for another sensor,*J*, if*J*falls in the sensing range of*I*, the sensor senses in this region and J retreats. If*$d\left(I,J\right)\geq \;\frac{r}{2}$*,*I provides coverage in three overlapping sectors. Otherwise, it covers five overlapping sectors.

We present a methodology of sponsoring the coverage for overlapping groups; e.g., groups of sensors colored red and green (cf. Figure 6). In this case, the sensors of a group that is formed first sponsor the sensing coverage to the sensors of other group. In other words, the group appears to be formed to avoid overlapping with prior knowledge of the presence of other groups (this is why we named the algorithm GS-IoT which combines SSS and FCG). In Figure 6, the red color group is sponsoring coverage for the green color group. Since this process runs in slots, in the next slot, groups will be formed once again. This saves energy. Moreover, if a sensor finds the status engaged in HelloRep packets from all its neighbours, and these neighbours sponsor full coverage, then it sets its timer for the next slot and goes in sleep mode. Otherwise, it senses in the uncovered region. Sensors always broadcast a confirmation report to their surroundings (when they go into engaged or ready state from busy state). The SSS algorithm which implements this scenario for sponsoring coverage is presented below (see Algorithm 2). As we are taking a large number of sensor nodes, we are able to ensure that every sensor has at least two other sensors in the sensing range. The number of sensors required for this purpose could be obtained by Equations (5) and (6). Once ensured, GS-IoT framework starts working. The framework works in slots. Each slot is divided into two different phases: FCG and SSC.

We do agree that fixed network infrastructure for information dissemination enabled by Software Defined Networking (SDN) architecture can play a significant role [39]. It can support IoT based smart service centric networking particularly for better performance and energy balancing oriented energy conservation. However, the deployment and maintenance cost of such a fixed network infrastructure is higher than the smart IoT networking environment. The proposed GS-IoT framework is an integrated network architecture featuring smart information gathering as well as disseminating via major access points. Here, intelligent network operating functions as SDN can be implemented at the edge, which is more practical considering growing IoT scenarios around the world for enabling smart environments.

### 3.4. Complexity Analysis

The complexity of the proposed GS-IoT framework can be majorly defined on the basis of two distributed algorithms including fast coverage grouping (FCG) and sponsoring aware sectorial coverage (SSC). The grouping is majorly relying on the number of sensors in the distributed group represented by K, and the waiting time of each group member represented by W. Therefore, the execution time complexity of the overall grouping procedure can be represented as O(K × W). Similarly, the sectorial coverage Algorithm 2 execution time can be defined in terms of numbers of sensor pairs in the sensing region group G, and the size of set of sectors represented by V. This can be represented as O(G/2 × V). These operating functions of the proposed framework executed sequentially and distributed in the manner in the scaled IoT network environment; therefore, the overall complexity of proposed framework can be defined as O(G/2 × V) assuming the sectoring computationally higher than the grouping operations which is majorly based on group size considered to be approximately 6–8 sensors.
**Algorithm 2: Sponsoring Aware Sectorial Coverage (SSC)**$G_{i\;}$*: ith group in sensing region. J, K and L: Group members**V: set of sectors to be avoided by present sensor**A: current set of sectors to be avoided**e (j): number of response with status engaged to sensor j**E (j): set of engaged sensors which responses**I:  set of sectors avoided intra group by*$i^{th}$ sensor.***begin:******for** (all combination of J, K, and L taking two together)****if*** (*d (J, K)*<r/2**)**         /*sponsoring 5 sector coverage to each other*/*randomly decide for sponsoring the sensing region (Bernoulli distribution)****else if****(d (J, K)*≥r/2)        /* sponsoring 3 sector coverage to each other* /*randomly decide for sponsoring the sensing region (Bernoulli distribution).***else**         /* sponsoring no sector coverage* /*do nothing****end if******end for******for****all member j*$\in \;G_{i}$     /* checking for external sponsoring*/
**if**(e (j) > 0)     /* it is sponsored by other external node*/V= { };        /*take an empty set for collection of sectors to be avoided*/***for** i= 1:1: e (j)**V= V*$\;\cup^{\;}A$***end for******end if******if****(V*$\;\cup^{\;}I$is contiguous) /* at a time sensor can have only one orientation, contiguous */*avoid sensing in sectors*$\in \left(V\;\cup^{\;}I\right)$***else****sense as if there is no sponsoring externally****for** (all members j of E (j))*send message of not taking sponsorship /*others take advantage of sponsorship*/***end for******end if******end for******end begin***

## 4. Performance Evaluation and Analysis of Experimental Results

### 4.1. Environmental Settings

In this section, a detailed explanation of experimental and simulation framework is presented, which is utilized for the performance evaluation of GS-IoT and comparative investigation with existing recent and relevant literature. The technical significance and characteristics of the mathematical modeling of the proposal is evaluated as analytical analysis considering a different range of parameter settings for critical impact analysis. This analytical investigation is similar to what has been done in recent IoT centric previous frameworks [34]. The scalability of the GS-IoT proposal has been evaluated using a network simulator-based implementation with realistic campus IoT environment consideration, similar to what is presented during modeling of the framework. Considering the coverage and overlapping centric modeling of the proposal, up to 1500 sensor nodes were utilized for coverage analysis of the IoT centric network architecture, and up to 2500 sensors nodes were used for coverage overlapping analysis of the network architecture. The suitability of the proposal under resource constrained IoT nodes has been experimentally tested using Arduino based hardware implementation of the algorithm. Low power Bluetooth modules were used for wireless communication among Arduino nodes with refined or predefined coverage setting for each node to control overlapping and monitor coverage under campus laboratory environment. For benchmarking, some recent and relevant literature GC-IoT [32] and GC-WSN [33] were considered in comparative investigation of the coverage performance. GC-IoT was an extended leach enabled energy centric framework for IoT lifetime maximization, whereas GS-WSN was a heuristic based solution of energy optimization problem in sensor networks. However, both these frameworks lack coverage optimization centric green communication modeling for IoT environments. Detailed critical investigation of the literature is provided in the related work section.

The further detailed settings of the performance evaluation for both simulation and hardware based implementations are based on standard environment settings considered in existing implementations of related frameworks in IoT environments. A three-dimensional sensing space is considered by deploying sensor nodes on both grounds and some walls of smart campus environments. Sensor nodes are considered to be of equal capability in all aspects such as initial energy and processing capacity. Transmission range and sensing range of each sensor nodes are considered to be 40 m. The fading exponent employed in sensing and transmission is assumed to be 2 in the statistical modeling. The maximum number of sectors of a sensor node are assumed to be 8 for grouping and sectoring. The maximum number of sensors in a group is considered 3 for overlapping control in coverage. The sensors are deployed according to Poisson distribution in the sensing field for continuous monitoring of the smart campus environment. It is also assumed that sensors follow the Poisson process while generating packets for communication. The simulations were performed for evaluating sensing coverage, sensor scheduling, rate of overlapping, and life span of the network architecture in the proposal. We have tested and validated our algorithms using a simulator implemented using C++ and Arduino based programing environment. The Monte Carlo simulation method is employed to analyze the sensing coverage area under realistic sensor deployment for IoT environment. Some further details of performance evaluation parameters are provided in Table 1.

Towards validating our analytical assumptions, we have performed simulation and hardware based experimental studies. We do agree that the shape of the physical sensing environment is constrained on the geographical scenario and the linear vehicular network environment. However, we want to highlight that our proposed framework is for such hybrid IoT network scenarios. Here, strategically deployed IoT sensors and campus vehicular networks form a holistic network environment and cooperate effectively for enabling smart services. This integration of networks significantly improves the network performance due to the linear vehicular network being part of the strategically deployed IoT networks in the campus environment.

### 4.2. Analytical Result Discussion

**Figure 7:**
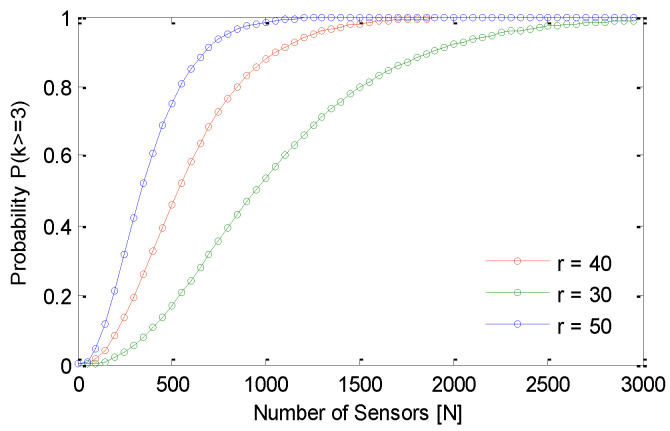
Probability distribution for P (k >= 3).

In Figure 7, we plot the probability of at least three sensors in any sub region of radius r/2. We take a realistic campus area for sensing region simulation. It should be noted that, for *r* = 40, N = 1500 the desired probability of having at least three sensors in a sub region is achieved. Once we have ensured the required density of sensors in the sensing region, we have to schedule these sensors for minimal overlapping. This minimal overlapping problem is comparable to a cover set problem that is proved to be NP- complete; however, we provide approximate solutions for this problem in Section 5. This algorithm ensures minimum overlapping and balanced energy consumption in different sensors. In this section, an analysis of convergence of GS-IoT is presented. We claim that GS-IoT converges fast. Since it is based on time of response from different sensors, these waiting times must be finite and small. In the simulation, a sensing field of similar area is assumed. Sensors are considered to be of equal capability in all aspects. Transmission range of each sensor is considered to be 50 m. Sensing ranges of a sensor are considered to be 30 m, 40 m, and 50 m. The fading exponent is assumed to be 2. The maximum sectors of a sensor are assumed to be 8. The number of sensors in a group is 3. In addition, 1500 sensors are deployed according to Poisson distribution in the sensing field.

**Figure 8:**
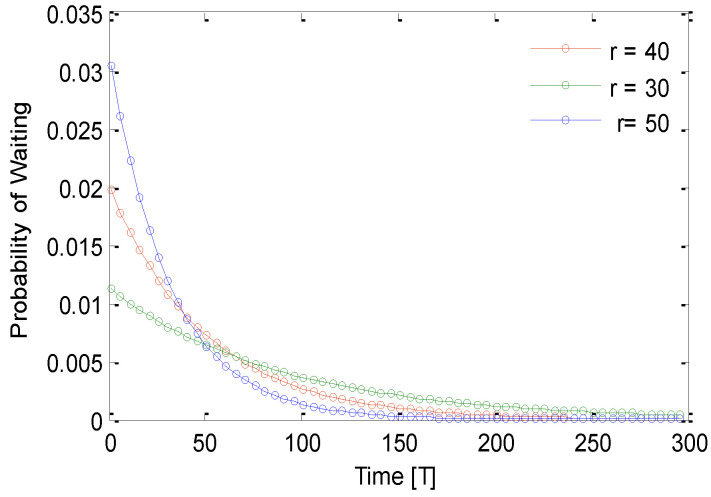
Probability of waiting for a finite time for the HelloReq packet.

**Figure 9:**
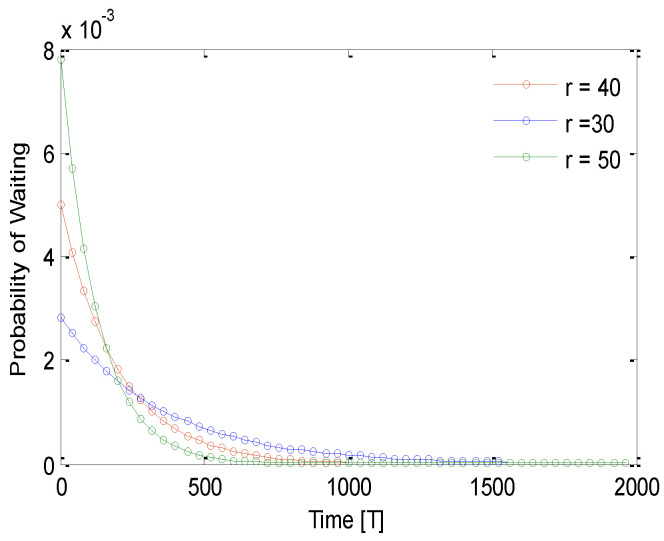
Probability of waiting for a definite time for sending the HelloRep packet.

Figure 8 shows the probability of waiting time for different values of time for different sensing ranges. The probability of waiting time decreases with an increase of sending a HelloReq packet. For example, for 50 s finite time, the probability of waiting is 0.007 for all selected sensing ranges. This decreases to 0.002, 0.005, and 0.006 for the for the sensing range 50 m, 40 m, and 30 m, respectively. It is noticeable that the probability of waiting before sending HelloReq packets decreases rapidly. This suggests that the process of grouping finishes in a small amount of time. Figure 9 shows the probability of waiting for different values of time for different sensing ranges. The probability of waiting time decreases with an increase of replying to a HelloRep packet. For example, for 200s finite time, the probability of waiting is 0.0018 for all selected sensing ranges. This decreases to 0.0002, 0.0004, and 0.006 for the sensing range 50 m, 40 m, and 30 m, respectively. It is noticeable that the probability of waiting before replying to HelloRep packets decreases rapidly. This suggests that process of grouping finishes in a small amount of time.

**Figure 10:**
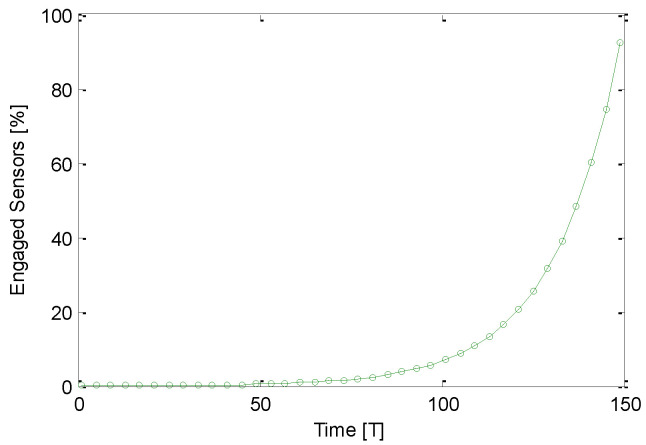
Engaged sensor % with in GS-IoT.

**Figure 11:**
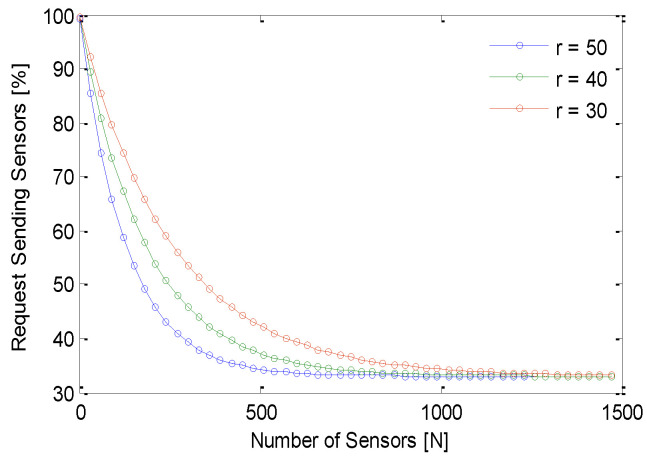
Requesting sensor % convergence in GS-IoT.

Variation of the percentage of engaged sensors with time is shown in Figure 10. The GS-IoT simulated for N=1500 sensors with a sensing range of r=40 m. No sensor is able to form a group up to a certain time T=50 s, and this is due to the fact that sensors initially attempt to form their own group of three sensors after a finite amount of time. An additional finite amount of time is spent in dialogue among neighbouring sensors to form the groups. GS-IoT starts making groups gradually after 50 units of time. After T=100, the number of engaged sensors increases very fast. It is noticed that GS-IoT forms groups rapidly. Figure 11 shows that GS-IoT is lightweight. Weight refers to number of transmissions required for group formation. We simulate the GS-IoT to estimate a percentage of request sending sensors as a function of a number of sensors considering the sensing range as a parameter. As the number of sensors increases, the percentage of request sending sensors decreases. For example, it converges about 35%. This is because of small group size. Since the time for grouping for different sensors are distributed exponentially, the probability of group formation is very high for all requests generated. This is why communication overhead to form the groups is considered minimal.

### 4.3. Simulation Results Discussion

**Figure 12:**
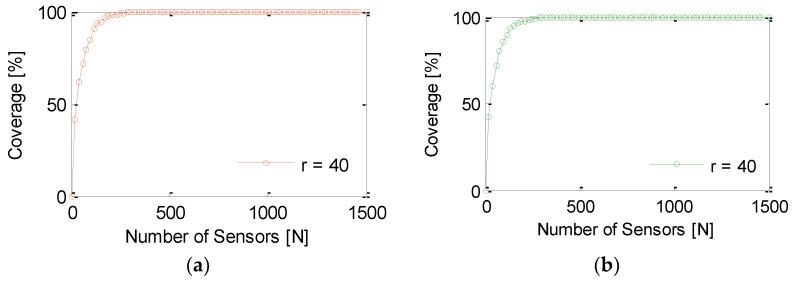
Comparison of coverage provided by proposed framework and literature (**a**) GC-IoT; (**b**) GS-IoT.

**Figure 13:**
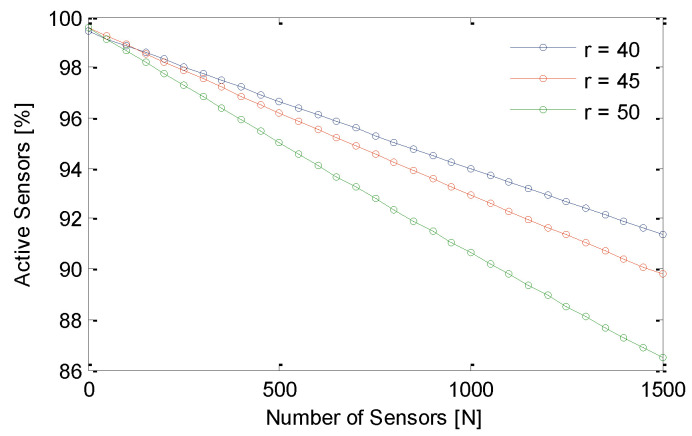
Performance of GS-IoT framework in setting sensor nodes off when in coordinated groups.

This section analyzes the performance of GS-IoT in the presence of redundant sensors. Here, redundancy is not with respect to the whole sensing region; rather, it exists in a specific zone. For the simulation, the transmission range of each sensor is considered to be 50 m. Sensing ranges of a sensor are considered to be 30 m, 40 m, and 50 m. In GS-IoT, there is a provision for setting the sensors off if they have been provided full sponsorship from other group sensors. Figure 12a,b show the performance of GS-IoT and omnidirectional sensing (ODS). It is clearly visible from the figure that GS-IoT preserves the coverage of what could be otherwise achieved through ODS. This is due to the fact that GS-IoT sets sensors off only when it confirms that coverage provided by a sensor is sponsored by other group members. Additionally, intra group coverage is provided interchangeably by member sensors. In the long run, both of these strategies perform almost interchangeably from the perspective of the coverage. Figure 13 shows the percentage of active sensors out of sensors deployed. For sensing range r=40 m and for the number of sensors, N=1500, approximately 9% of sensors are switched to sleep mode. For sensing range r=50 m, this percentage is 13. Even after GS-IoT has sent a significant number of sensor nodes in sleep mode, coverage is unaffected.

**Figure 14:**
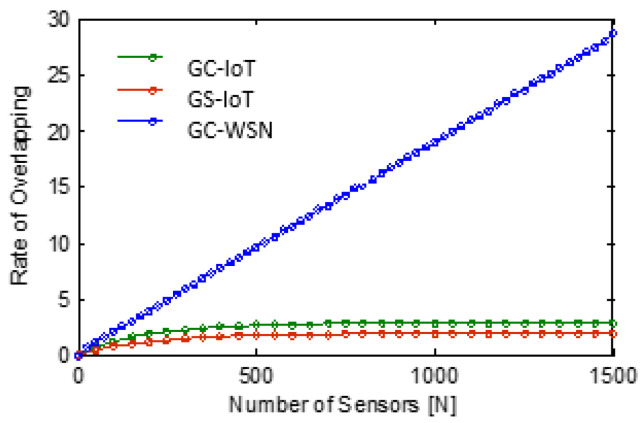
Overlapping rate comparison.

**Figure 15:**
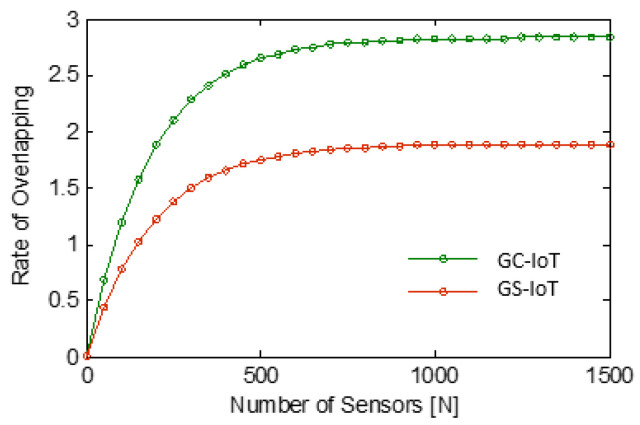
A better difference visibility of overlapping rate.

What we intended to do in this paper is to minimize overlapping. We see overlapping as the most visible wastage of energy. This energy saving enhances the overall life of network. GS-IoT does this efficiently. In Algorithm 2 of GS-IoT, a provision is provided to minimize overlapping in a novel way. It is based on sectorial sensing and sectorial transmission. It minimizes overlapping at two levels. At first, it stops intra-group overlapping. At the second level, it stops inter-group overlapping. Simulation studies with GS-IoT, GC-IoT, and GC-WSN were performed for a rate of overlapping in the entire sensing region. A rate of overlapping K signifies that each point in the entire sensing region is covered by K sensors on average, and this is different from k- coverage, which says that each point is covered by at least k sensors. Simulation results of the three sensing algorithms are shown in Figure 14. We observe that, for N=1500 and sensing range r=40 m, the rate of overlapping is about 29 for GC-WSN. It is about 2.9 in the case of GC-IoT and 1.8 for GS-IoT. The rate of overlapping remains constant for a higher number of sensors for GC-IoT and GS-IoT. The rate of overlapping is less for GS-IoT as compared with GC-IoT and GC-WSN. This is because of a GS-IoT employed sponsored aware sectoring coverage algorithm. This aspect is clearly visible in Figure 15. GS-IoT reduces the overlapping by a factor of 1/17 times that of GC-WSN and the reduction factor in comparison to GC-IoT is 5/8. It is clear that GS-IoT outperforms GC-IoT. This is because of elimination of intra group overlapping by GS-IoT.

**Figure 16:**
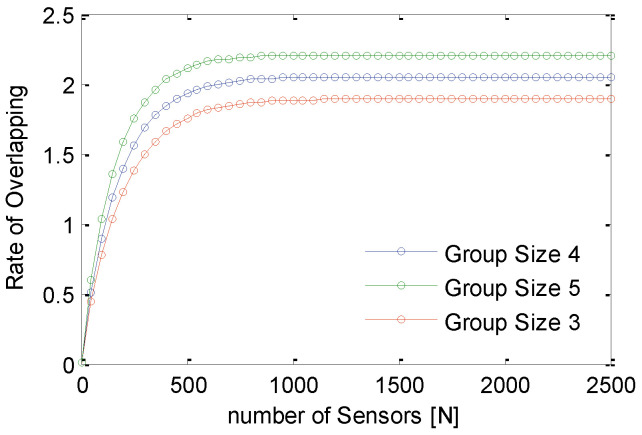
Overlapping rate of GS-IoT with group sizes.

**Figure 17:**
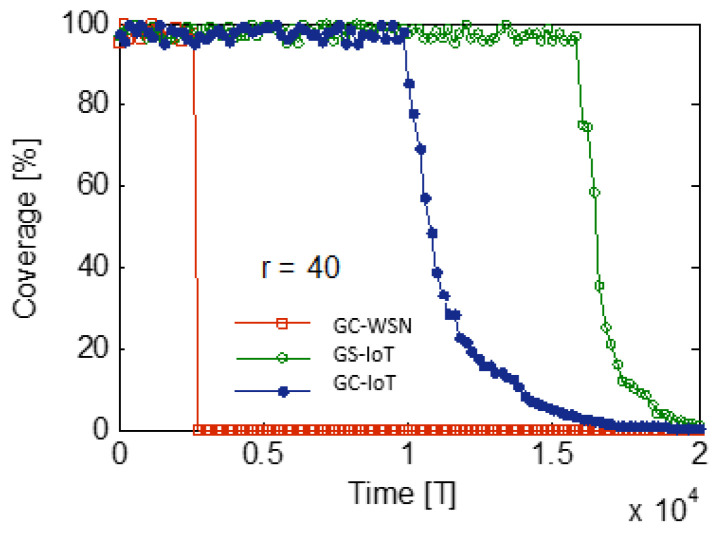
Life span comparison with literature.

Figure 16 shows performance analysis of GS-IoT for different group sizes. As the size of group increases, intra and inter group overlapping also increase. When a non-contiguous set of sectors is to be avoided, GS-IoT works as if there is no external sponsoring. As group size increases, the number of non-contiguous sets of sectors increase, and thus the overlapping. It turns out that the optimum size of a group is 3. To characterize the lifetime of the network, we consider a simplified energy model. In this energy model, we divide time into discrete slots. GS-IoT runs in slots and, after each slot, we measure the percentage of sensors that have not depleted their energy. We assume that sensors can perform four tasks in each slot in this energy model. First, they may be transmitting the data. Second, they may be receiving data. Third, they could be in sleep mode. Fourth, it is sensing the region. In this simulation, the following assumptions are made. For transmitting the data, 0.005 units of energy are consumed per unit of time. For sensing in one sector, 0.003 units of energy are consumed per unit of time. Energy consumption is 0.0001 units per unit of time in sleep mode. In addition, a sensor consumes energy 0.0045 units per unit of time for reception of data. Initially, each sensor starts with equal energy of 100 units. GS-IoT is simulated for 20,000 units of time. The simulation time is divided into 100 slots. In each slot, there are four rounds. Simulation results for life span in terms of coverage for GS-IoT, GC-IoT, and GC-WSN are shown in Figure 17. It is observed that, for sensing range r=40 m, the life span of the network in case of GS-IoT increases by about 1.54 times that of GC-IoT. It is 6.8 times that of GC-WSN. This is due to the fact that GS-IoT switches redundant sensors into sleep mode and reduces the overlapping. It is evident that GS-IoT improves the life span of a sensor network.

### 4.4. Hardware Result Discussion

**Figure 18:**
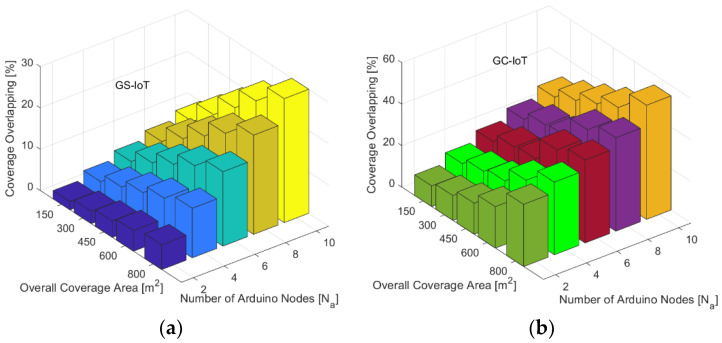
Hardware node-based coverage overlapping comparison (**a**) GS-IoT; (**b**) GC-IoT.

A hardware implementation of the GS-IoT framework was carried out using Arduino based Bluetooth low power enabled sensor nodes in a smart campus environment [40,41]. Ten hardware nodes were added gradually as an overall coverage area in such a way that effective communication was possible [42]. The nodes were brought closer for measuring coverage overlapping with each other considered while running the proposed framework on each node and the GC-IoT framework on the next separate round of implementation. The measured coverage overlapping rate is presented for comparative result analysis in Figure 18a,b. It can be observed that the coverage overlapping rate is considerably lower in the case of a proposed framework as compared to the literature in consideration. In particular, the coverage overlapping rate gradually increases with an increasing number of hardware nodes. It is less than 20% for up to six hardware nodes and reaches approximately 30% with 10 hardware nodes and 800 m^2^ coverage area. This reduced overlapping significantly improves the energy performance of the network in terms of longer network lifetime for hardware nodes. This can be attributed to the effective grouping and sectoring of hardware nodes for providing coverage while they gradually join the network in the proposed GS-IoT framework. However, in the case of considered literature CG-IoT, the coverage overlapping rate is significantly higher. Specifically, it is up to 36% with six hardware nodes and gradually reaches up to 56% coverage overlapping with 10 hardware nodes and 800 m^2^ overall coverage area. The greater overlapping rate is due to the interference centric modeling where nodes might have considerable overlapping coverage area while not interfering each other communications up to certain level. Further, in GC-IoT, local clustering and sectoring were not materialized in the modeling of the framework which is responsible for higher coverage overlapping with larger overall coverage area and increasing number of hardware nodes as compared to the proposed framework.

### 4.5. Summary of Experimental Observations

Major findings of experimental study considering analytical, simulation, and hardware investigation are listed below:

- GS-IoT increases the life span of network about 1.54 times that of GC-IoT, as it lacks intra-group and inter-group overlapping prevention policy.
- The coverage overlapping rate gradually increases with an increasing number of hardware nodes. It is less than 20% for up to six hardware nodes and reaches approximately 30% with 10 hardware nodes.
- As the number of sensors increases, the percentage of request sending sensors decreases. For example, it converges about 35%. This is because of small group size.
- For sensing range r=40 m and for number of sensors, N=1500, approximately 9% of sensors are switched to sleep mode. For sensing range r =50 m, this percentage is 13.
- We observe that, for N=1500 and sensing range r=40 m, the rate of overlapping is about 29% for GC-WSN. It is about 2.9% in case of GC-IoT and 1.8% for GS-IoT.

## 5. Conclusions and Future Work

In this paper, we have presented GS-IoT, an efficient algorithm that consists of two algorithms FCG and SSC to minimize the overlapping of sensing coverage and maximize the life span of wireless sensor networks. A novel idea for grouping of sensors is presented in FCG. This algorithm is very suitable in complex, dynamic, and self-organizing networks where an entity is highly prone to failure. FCG randomizes the process of grouping in each slot to make the network fault tolerant. Moreover, it avoids the communication overhead when the sensors send requests to form groups. SSC reduces the intra-group and inter-group overlapping using the sectorial sensing model. It sets off redundant sensors to save energy preserving the sensing coverage. Simulation results show that the GS-IoT outperforms the GC-IoT in terms of life span and rate of overlapping. GS-IoT increases the life span of the network about 1.54 times that of GC-IoT, as it lacks an intra-group and inter-group overlapping prevention policy. The success of the coverage problem depends on the level of cooperation in the sensors, and there could be many schemes to establish this cooperation. We have proposed one such scheme. In future works, the extension of GS-IoT framework will be explored focusing on an 5G centric IoT application using next generation wireless advancement such as MIMO and SWIPT. Its applicability in the indoor IoT environment particularly in energy conservation considering the higher interference and related architectural and protocol level changes will also be the quest in the future.

## Figures and Tables

**Table 1:** Major simulation parameters.

Simulation Parameter	Value Considered in Simulation
Sensing fied as campus	1500 m^2^
Radius of tranmission (R^t^)	40 m
Radiusof sensing in nodes (R^s^)	50 m
Initial enery in sensor nodes (E^i^)	5j
Network lifetime of nodes	1st no event report
Number of sensor nodes	1500–2500
Number of sink nodes	30–40
Energy expenditure (E^ele^)	40 nj/bit/signal
Length of data packet (header and payload)	4000 bits
Exponent of pathloss (Φ)	2
sensing packets size	2000 bits
Data agregation energy	5 nj/bit/signal
Grouping size of sensors	6–8
Overlapping factor	2–3
